# The impacts of product characteristics and regulatory environment on smokers’ preferences for tobacco and alcohol: Evidence from a volumetric choice experiment

**DOI:** 10.1371/journal.pone.0320023

**Published:** 2025-03-12

**Authors:** Shaoying Ma, Ce Shang, Vuong V. Do, Jidong Huang, Terry F. Pechacek, Scott R. Weaver

**Affiliations:** 1 Center for Tobacco Research, The Ohio State University Comprehensive Cancer Center, Columbus, Ohio, United States of America; 2 Department of Internal Medicine, The Ohio State University Wexner Medical Center, Columbus, Ohio, United States of America; 3 Cardiovascular Research Institute, School of Medicine, University of California, San Francisco, California, United States of America; 4 Department of Health Policy and Behavioral Sciences, School of Public Health, Georgia State University Atlanta, Atlanta, Georgia, United States of America; 5 Department of Population Health Sciences, School of Public Health, Georgia State University Atlanta, Atlanta, Georgia, United States of America; Xi’an Jiaotong University, CHINA

## Abstract

**Objective:**

Concurrent use of alcohol and cigarettes is well-documented in the literature. However, it is unclear how e-cigarette regulations in a growing number of localities impact the use of tobacco and alcohol in the US. This study aims to evaluate the impacts of excise taxes, tobacco use restrictions in restaurants/bars, and availability of alcohol flavor in e-cigarettes on tobacco consumption, and their cross impacts on alcohol consumption.

**Method:**

A total of 181 US adult smokers who were using e-cigarettes and consuming alcohol participated in online volumetric choice experiments and reported on the quantity they would purchase among cigarettes, closed-system e-cigarettes, beer, and one other alcohol product (wine/liquor) under varying policy scenarios.

**Results:**

Estimated own-price elasticities of demand for beer, liquor/wine, and cigarettes were -0.10, -0.11, and -0.16, respectively (*p* <  0.001). Higher beer (cross-price elasticity =  0.13) and liquor/wine prices (cross-price elasticity =  0.05) increased e-cigarette consumption (*p* <  0.05). If e-cigarettes were allowed in bars/restaurants, their consumption increased by 2.4 units (*p* <  0.001), and if cigarettes were allowed in bars/restaurants, e-cigarette consumption increased by 1.9 units (*p* <  0.01), relative to the mean consumption level. Greater reported weekly spending on alcohol and/or tobacco was associated with higher consumption of all products (*p* <  0.001).

**Conclusions:**

Higher taxes or prices may reduce the consumption of beer, liquor/wine, and cigarettes. E-cigarettes are economic substitutes for alcohol among smokers who are currently drinking and using e-cigarettes. Regulating tobacco indoor use will have an impact on e-cigarette consumption.

## Introduction

Research has established a strong relationship between tobacco use and alcohol drinking at the population, environmental, individual, and biological levels. First, tobacco and alcohol use may reinforce each other [1]. Studies have shown that ethanol, and therefore all types of alcoholic beverages, increases smoking among people with alcoholism [2], and that smokers and e-cigarette users showed increased risks of harmful alcohol use compared to non-smokers and non-e-cigarette users [3–6]. In addition, drinking in bars is consistently associated with high smoking rate among young adults regardless of tobacco control policies [7]. Second, tobacco companies often linked cigarettes with alcohol in their marketing and promotion (e.g., tying cigarette and alcohol purchases together) to prolong the use of both substances [8]. As a result, concurrent use of tobacco and alcohol has been widely documented in the literature [9–12]. which further shapes the use behaviors of both products [4,13–15], such that attempts to quit smoking are often hindered by concurrent alcohol use [16–20].

Given the relationship between alcohol and nicotine/tobacco products, policies aimed at reducing the use of one substance may have a spillover or cross-product effect on the other. For instance, increasing cigarette taxes and imposing restrictions on smoking in public places such as restaurants and bars have been found to reduce excessive drinking and associate with a reduced likelihood of alcohol consumption [21–25]. However, the majority of studies examining the link between tobacco use and alcohol consumption have primarily focused on cigarettes and beer, which may limit their relevance to the current tobacco use landscape in the US, where e-cigarettes have overtaken cigarettes as the most used nicotine/tobacco product among youth and young adults [10,26].

So far, there is limited research on individual persons who use both e-cigarettes and alcohol. It is unclear how the consumption of alcohol, together with traditional cigarettes and e-cigarettes, impact tobacco use outcomes and trajectories among people who both smoke cigarettes and vape e-cigarettes. Additional knowledge gaps also exist regarding the effect of e-cigarette regulations, which are often less strict than those for combustible cigarettes (e.g., lower excise tax incidence, permitted to use indoors, etc.), on alcohol consumption [27–29].

Another link between e-cigarettes, cigarettes, and alcoholic beverages has been the availability of e-cigarette flavor options resembling alcoholic beverages (e.g., wine, liquor) [30]. Unlike cigarettes that are either menthol or tobacco flavored, e-cigarettes come with a wide range of flavors including alcohol flavors, through which the concurrence of alcohol drinking and tobacco consumption may be strengthened [31]. Alcohol is also a common flavor option in cigars, little cigars and cigarillos [30,32–34]. Alcohol flavored e-cigarettes are popular among users and widely available in the US market. 21% of adult US e-cigarette users in 2015 reported using alcohol or coffee flavored products [35]. An evaluation of the e-cigarette online market in 2022 further demonstrated that 17% of e-cigarettes sold online contained alcohol flavors [36]. Recognizing the role of appealing flavors such as alcohol flavors in e-cigarette use, an increasing number of states and localities in the US ban the sales of flavored e-cigarettes, including alcohol flavors [37,38]. While these bans may have reduced vaping, none of the studies specifically examined alcohol flavor availability in e-cigarettes and how that may influence the choices among tobacco and alcohol [39], which is a clear gap in the context of cross-product policy impacts.

To fill in these gaps, this study conducted a volumetric choice experiment (VCE) to examine how excise taxes, the availability of alcohol-flavored e-cigarettes, and indoor use restrictions on tobacco may influence individuals’ stated consumption of alcohol (beer, wine, liquor), e-cigarettes (disposables and refillable cartridges), and cigarettes among adults who reported currently using alcohol, e-cigarettes, and cigarettes. As an extension of discrete choice experiments (DCEs), VCEs allow for an evaluation of consumption levels in addition to product choice (i.e., to buy or not to buy), thereby having the advantage in assessing the co-use and poly-use behaviors. As a novel method, VCEs have been increasingly used in recent years to investigate policy impacts on cigarette [40–44], waterpipe tobacco [40,42,45] and e-cigarette [41,43,44] use. However, this method has not been used to evaluate cigarettes, e-cigarettes, and alcohol systematically.

Specifically, we tested whether regulations banning e-cigarette/cigarette use in restaurants and bars and removing alcohol flavors in e-cigarette products would be associated with a decrease in alcohol, cigarette and e-cigarette consumption. In addition, with varying taxes and prices, we were also able to estimate the own- (i.e., how the price changes of a product impact its own use) and cross-price elasticities (i.e., how the price changes of a product impact the use of another product) among these products. A positive cross-price elasticity suggests substitution, whereas a negative cross-price elasticity suggests complementarity. Although there is rich literature showing that e-cigarettes and cigarettes are likely economic substitutes [46–50] and cigarettes and beer are likely complements, there has not been any studies, to our knowledge, expanding the analysis to a multiple-product system that is inclusive of e-cigarettes, cigarettes, and various alcoholic beverages. Our study is the first to systematically evaluate the economics relationships among these products [40,51].

The finding from this study could provide important empirical evidence in shaping local, state, and federal policies regulating tobacco and alcohol products, including the design of excise taxes, implementation of tobacco indoor use restrictions, and ban on alcohol-flavored e-cigarettes. A unique contribution of this study is accounting for product heterogeneity (different tobacco and alcohol products) and the focus on how policies regulating one product hypothetically impact the consumption of another, providing novel experimental evidence that addresses confounding issues in previous studies using survey data or retail sales data [46–50].

## Materials and methods

### Participants & survey procedures

This study was approved by the Georgia State University Institutional Review Board. Informed consent was given for participation in the research. The recruitment period started on December 8, 2020, and ended on October 12, 2021. Consent was provided on the computer screen, and study participants indicated their consent by moving to the next screen. We received consent for them to complete the screener/eligibility survey and then again for the full study at the baseline survey.

We recruited participants who reported past 30-day alcohol consumption for an alcohol-tobacco co-use study from the parent Adult Consumers of Tobacco Study (ACTS), which was designed to examine the contextual, individual, and product factors that affect smoking and e-cigarette use patterns during the period immediately following e-cigarette initiation. Eligibility criteria for the parent ACTS study were: 1) be at least 21 years old; 2) have the ability to read and comprehend English; 3) have either recently (within the past 30 days) started using e-cigarettes or re-initiated e-cigarette use after abstaining for at least one year; 4) have smoked a minimum of 100 cigarettes in their lifetime; and 5) either currently smoke cigarettes some days or every day, or have smoked within the past 60 days. The study participants for ACTS were recruited nationally using targeted online and social media advertisements (e.g., Facebook/Instagram, Craigslist, Google Ads, Twitter) from December 2020 through October 2021 [52]. A total of 303 participants were recruited for ACTS and completed the baseline survey on tobacco use, past 30-day alcohol use, and demographics.

The 193 participants who reported past 30-day consumption of alcohol were invited to complete a supplemental survey with VCE focused on tobacco and alcohol co-use 3 days following completion of the baseline survey. This sample provides a unique opportunity to study e-cigarette and tobacco users who are at a transition stage between products with different relative harms, likely providing a window for behavioral changes in multiple substance use, such as drinking. As the participants were smoking cigarettes and consuming alcohol, and recently started to or resumed e-cigarette vaping, they were also facing tradeoffs among various products and thereby suitable candidates for choice experiments. In addition, this is a sample of participants who had experience using all three product categories: alcohol, cigarettes, and e-cigarettes and were therefore less likely than other population groups to provide biased responses to hypothetical choice tasks. Of those eligible, 182 participants (94% of those invited) completed the VCE survey within a 48-hour window [53]. Participants were provided $25 for completing the baseline assessment and $15 for completing the VCE survey in the form of e-gift cards. This research study was designated exempt by the Georgia State University Institutional Review Board.

Twenty individuals participated in a pilot of ACTS, which contained the screener, baseline survey (including its tobacco-only VCE, and the display and instructions for which the alcohol-tobacco co-use VCE in this study were based), and several weekly surveys. Although the pilot did not include this tobacco-alcohol co-use VCE, ACTS participants had the opportunity to become familiar with choice tasks from the baseline survey prior to the tobacco-alcohol co-use survey. In addition, after some participants completed the baseline and tobacco-alcohol co-use surveys, we contacted them to inquire about their understanding of the choice tasks, and subsequently updated the instructions and programming in this tobacco-alcohol co-use VCE.

### Volumetric choice experiment design

In this VCE, we used a labeled design with four products that were always presented to participants: cigarettes, beer, e-cigarettes, and one other alcoholic beverage (Table 1). For e-cigarettes and one other alcoholic beverage, we manipulated product types so that there are two types of e-cigarettes: rechargeable with cartridges vs. disposables, and two types of alcoholic beverage (in addition to beer): liquor vs. wine.

**Table 1:** Product types, attributes, and levels.

Product	Product type	Product attribute	Attribute levels
Tobacco	Regular cigarettes	Price (tax burden) Flavor Restaurant/bar restrictions	Price per pack of 20 cigarettes with 25%, 50%, or 75% tax burden on retail prices) Tobacco, menthol Allowed vs. not allowed
	E-cigarettes: rechargeable pod with pre-filled cartridges vs. disposable pod	Price (tax burden) Flavor Restaurant/bar restrictions	Price per unit (with 0%, 25%, 50%, or 75% tax burden on retail prices) Tobacco, menthol, alcohol, fruit Allowed vs. not allowed
Alcohol	Beer	Price (tax burden)	Price per 12 oz can or bottle (with 0%, 25%, 50%, or 75% tax burden based on retail prices)
	Alternative alcohol beverages: table wine vs. distilled liquor	Price (tax burden)	Price per 5 oz glass (with 0%, 25%, 50%, or 75% tax burden based on retail prices)

Attributes and their corresponding levels were chosen based on policy interests, including various tax burdens based on final retail prices (i.e., excise taxes as a percentage of tax inclusive retail prices), flavors, and tobacco indoor restrictions. Specifically, taxes aimed at raising prices are the most widely adopted regulatory policies for alcohol and tobacco, and the tax burdens, defined as taxes as a percentage of retail prices, are used by policymakers to measure policy strength and set guidelines for products that have negative public health consequences [29,54–56]. Furthermore, tax burdens are compared across substances or products (e.g., cigarettes vs. e-cigarettes) to ascertain which products are taxed higher to guide a systematic approach to tax multiple products [29,57]. Therefore, we choose a range of tax burdens ranging from 0% (i.e., no excise taxes) to 75% of retail prices (25%-75% for cigarettes because all of U.S. states tax cigarettes), which reflects the existing and possible levels of tax burdens. In addition, a 75% tax burden was chosen because the World Health Organization (WHO) Framework Convention on Tobacco Control (FCTC)’s recommendation that the cigarette tax burden should be set at 75% of retail prices [58–60].

Next, we used the varying tax burdens and market prices to determine the final prices shown in the choice experiments. The marketplace prices also incorporate regional differences as much as possible. Specifically, for beer, liquor, and wine (Northeast, Midwest, South, West), we used regional-prices estimated using the 2019 Economist Intelligence Unit (EIU) city-level price data [59]. For cigarettes, we used the state level prices from the *Tax Burden on Tobacco* [60]. For e-cigarettes, we used the prices of e-cigarettes listed by leading brands (JUUL and Blu) online at the time of the study [61]. We used these price estimates for all states because when we examined Nielsen Retailer Scanner data, we found the variations in the retail prices of e-cigarettes by states to be limited. Moreover, the typical brands such as JUUL and Blu sold products nationally via online purchases and shipping. Therefore, we decided to use the prices listed by these leading e-cigarette brand vendors as the market prices for e-cigarettes.

In addition to tax burdens (prices), we also manipulated flavor restrictions and tobacco indoor restrictions for tobacco products, which are policies at the intersection of alcohol and tobacco (i.e., alcohol flavor in e-cigarettes may be banned, tobacco use may be restricted at alcohol-serving locations). To reflect these policy scenarios, the flavors for cigarettes were set to be either tobacco or menthol, while the flavor for e-cigarettes was set to be among tobacco, menthol, alcohol, or fruit. In the VCE, when displaying alcohol-flavored e-cigarettes, the alcohol flavor was shown as a digital icon of cocktail, and in the instructions to participants, we used the term “alcohol” along with the icon, as alcohol-flavors in nicotine/tobacco products can also be marketed as specific drinks such as “mojito” or “margarita” [30]. Tobacco indoor use status was depicted by either a “no smoking (vaping)” or “smoking (vaping) is allowed” at bars or restaurants pictograms with words. The participants are also allowed to choose not to buy any products, which is equivalent to the “opt-out” option in a discrete choice experiment setting.

**Fig 1:**
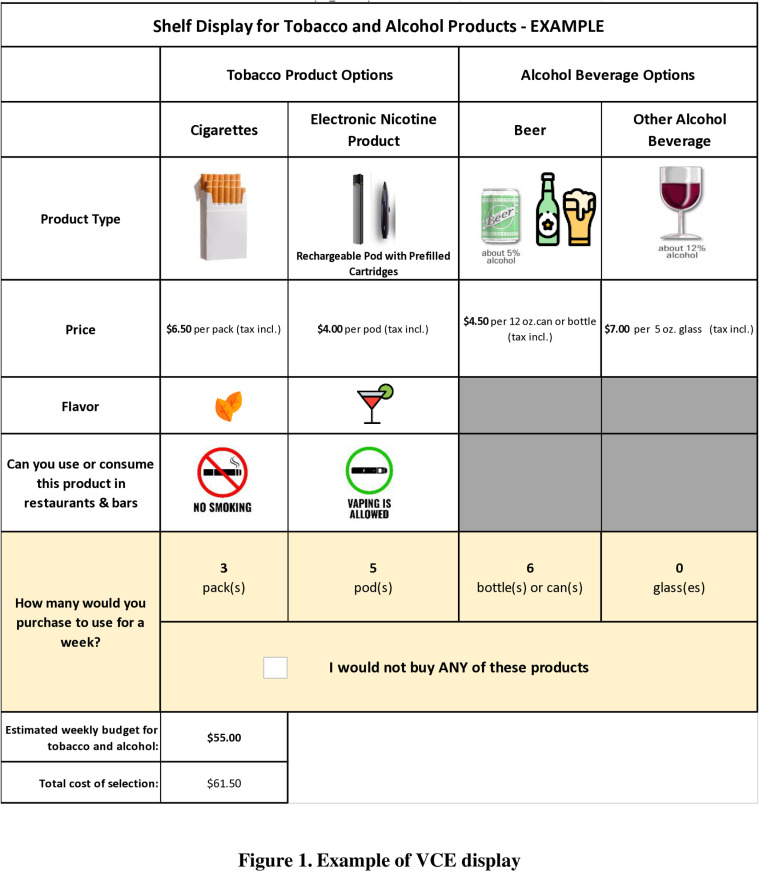
Example of VCE display.

In order to manage participants’ fatigue, we decided to present them 8 choice tasks based on prior experience and feedback from participants. Fig 1 shows an example of one choice task with policy scenarios. The VCE design results in (4*2*4*3*2) 192 potential combinations with additional constraints that forbid certain levels (e.g., flavors are not available for alcohol products). We then used Negene software and a D-efficiency design to choose one design with 16 choice tasks. Next, we divided the 16 choice tasks into 2 blocks that are different from each other and each contains 8 unique choice tasks [62]. Participants were randomized into one of the two blocks and answered 8 choice tasks (Fig 1 provides an example of one choice task).

Prior to the administration of the VCEs, participants were presented with an image displaying different types of alcoholic beverages and their corresponding standardized volumes to prepare them to compare prices across different beverage types. Next, we provided a detailed description and instructions for the VCEs, including the tobacco and product options that would be shown to them. We also used instructions to help elicit truthful answers, including the emphasis that participants should not consider saving, sharing, selling, or stockpiling these products for a later time.

Further, we asked participants to report their typical weekly budget for tobacco and alcohol and asked them to make purchases considering this budget. Participants were then presented with an example image of a completed choice task (Fig 1), accompanied by a description of the choice made. See “Instructions to participants about VCEs” in S1 File in S1Data for detailed instructions that were presented to the participants. Study participants were told there are no right or wrong answers, and they were asked to report what they would actually do if products available for purchase in the experiment were the only options. Participants were randomized to be shown the price as either “$x.xx (sales tax incl.) + $x.xx (excise tax) per [unit]” or “$x.xx per [unit] (tax incl.).”. An example of the latter price display format is shown in Fig 1.

### Measures: Outcome and demographic variables

Participants reported the quantity of each product shown in a choice task that they would purchase for consumption in a week. The products were measured in different but comparable standard units, including packs, pods, bottles or cans, and glasses or shots for cigarettes, pod e-cigarettes, beer, wine, and distilled liquor, respectively. As participants entered the quantities for each product, the total cost of their selection(s) would be updated and displayed in real time on their screens.

Prior to the VCE, each participant was asked “About how much did you spend per week on average on alcohol?” and they would enter dollars and cents. Similarly, they were also asked “About how much did you spend per week on average on tobacco or nicotine products in the past 30 days?” Their typical weekly expenditure on tobacco and alcohol was then calculated from those questions and displayed next to their total cost of selection. Participants reported gender, race, and ethnicity when completing the eligibility survey. The baseline survey assessed the highest educational attainment, family income in 2019 before taxes, sexual orientation, and current employment status.

### Analysis

The outcomes of VCEs (reported units of purchase) included a significant amount of 0s (no purchases). Therefore, we estimated the impacts of attributes on consumption, including own and cross-price elasticities, using zero-inflated negative binomial regressions and controlled for participant demographic characteristics. Standard errors were clustered at individual participant level. We also used generalized estimating equation (GEE) models and random-effects generalized least squares (GLS) regressions as alternative specifications to test the sensitivity of our study results.

To estimate own- and cross-price elasticities of demand as well as the impacts of non-price attributes (types, flavors, and public use status), we employed the following model specification for zero-inflated negative binomial regression:


$$\begin{array}{l} {\text{Consumption}}_{ist}=a_{0}+a_{1}{\text{OwnPrice}}_{ist}+a_{2}{\text{OtherPrice}}_{i\left(j!=s\right)t}+a_{3}{\text{Flavor}}_{ist}+a_{4}{\text{PublicUse}}_{ist}+\\ a_{5}{\text{ProductConstant}}_{ist}+a_{6}X_{i}+\varepsilon_{ist} \end{array}$$ (1)

In the above equation, *i* denotes individual participant, s denotes the product alternative, *j* denotes other products presented in the same choice task, and *t* denotes choice task; consumption is the number of units of alternative *s* that participant *i* chose to purchase in choice task *t*. OwnPrice_*ist*_ is the price of the corresponding product *s* (*a1* estimates own-price elasticities). OtherPrice_*i (j!=s)t*_ is a vector of prices of other products or alternatives that are presented in the same choice task *s* (*a*_*2*_ estimates cross-price elasticities).

Flavor_*ist*_ is a vector of indicators for flavor options of nicotine/tobacco products, i.e., tobacco or menthol for cigarettes, tobacco/menthol/fruit/alcohol for e-cigarettes, and not applicable for alcoholic beverages. PublicUse_*ist*_ indicates whether a nicotine/tobacco product (cigarette or e-cigarette) *s* is allowed to be used in bars/restaurants in choice task *t* for participant *i*, and not applicable for alcoholic beverages. ProductConstant_*ist*_ is a vector of indicators for whether the product is beer, cigarette, e-cigarette, and liquor/wine, respectively (1 yes and 0 no), which estimates the differences in product preferences. X_*i*_ are individual participants’ demographic characteristics, and *ε*_*ist*_ are the error terms. We also conducted sensitivity analysis by estimating tax burden effects instead of own- and cross-price elasticities.

Similarly, we used equations 2 and 3 to estimate the policy effects of tobacco indoor use and the availability of alcohol flavors in e-cigarette products, respectively, on both tobacco and alcohol consumption.


$${\text{Consumption}}_{ist}=b_{0}+b_{1}{\text{PublicUse}}_{i\left(j=tobacco\right)t}+b_{3}{\text{Price}}_{ist}+b_{4}{\text{Flavor}}_{ist}+b_{4t}+b_{6}X_{i}+\sigma_{it}$$ (2)

$${\text{Consumption}}_{ist}=c_{0}+c_{1}{\text{Flavor}}_{i\left(i,j=alcohole-cigarette\right)t}+c_{2}{\text{Price}}_{ist}+c_{3}{\text{PublicUse}}_{ist}+c_{4}{\text{ProductConstant}}_{is}+c_{6}X_{i}+\eta_{it}$$ (3)

We controlled for the following demographic variables in all specifications: age, gender, sexual orientation, race/ethnicity, education, employment status, and total family income (in 2019 before taxes). Participants with missing age (n =  1), before-tax family income in 2019 (n =  2), employment status (n =  1), or state of residence (n = 2) were removed from the analysis. In addition, 17 observations with extreme values of consumption (greater than 50 units) were also excluded. There were 5,131 observations from 169 participants remaining in the analytic sample. Choice experiments provide repeated measures from each participant, and thus our VCE study is designed to detect small effect sizes [63].

## Results

Table 2 and S3 Table in S1 Data present the summary statistics of participant demographics (N = 169), as well as information about their smoking, vaping, and alcohol use behaviors. The average age of study participants was 38. The majority of participants (>90%) reported current smoking and/or e-cigarette use with 66% reporting daily smoking while 44% were vaping daily. Among e-cigarette users, rechargeable pod systems were the most used while vape pens were least common. More than 62% reported consuming more than 2 alcohol drinks on a typical consumption day.

**Table 2:** Summary statistics of participant smoking and vaping status and alcohol use.

	Number of participants	Percent
**Smoking status**		
*Do you now smoke cigarettes*		
Not at all	16	9.47
Some days	42	24.85
Every day	111	65.68
*On how many of the past 7 days did you smoke cigarettes*		
None	16	9.47
1–3 days	23	13.61
4–6 day	21	12.42
7 days	109	64.5
*On average, on days you smoked, how many cigarettes did you usually smoke each day*		
<= 5	42	27.45
6–10	35	22.87
11–15	35	22.88
16–20	31	20.25
> 20	10	6.53
Refuse to answer	16	9.47
**Vaping status**		
*Do you now use e-cigarettes with nicotine*		
Not at all	11	6.51
Some days	83	49.11
Every day	75	44.38
*On how many of the past 7 days did you use e-cigarettes*		
None	16	9.47
1–3 days	48	28.41
4–6 day	36	21.3
7 days	69	40.83
*(Past 7 days) On average, when you used e-cigarettes, how often did you use it per day*		
At least once per day	7	10.45
Every once in a while throughout the day	25	37.31
Fairly frequently throughout the day	27	40.3
Almost always throughout most of the day	8	11.94
Refuse to answer	102	60.36
*Type of e-cigarette products you use most often*		
Cig-a-likes	27	15.98
Vape pens/eGo	22	13.02
Rebuildable/mechanical mod or box mod	35	20.71
Rechargeable pod systems	46	27.22
Disposable pod systems	39	23.08
**Alcohol use**		
*How many drinks containing alcohol do you have on* a *typical day when you are drinking*		
1 or 2	64	37.87
3 or 4	53	31.36
5 or 6	35	20.71
7, 8, or 9	9	5.33
10 or more	7	4.14
Refuse to answer	1	0.59
**Weekly spending on tobacco and alcohol**		
*(Past 30 days) About how much did you spend per week on average on tobacco/nicotine products*		
<= $15	27	15.98
$16 – $40	49	28.99
$41 – $75	56	33.13
> $75	37	21.88
*About how much did you spend per week on average on alcohol*		
<$5	41	24.25
$6 – $11	25	14.78
$12 – $20	36	21.30
$21 – $31	23	13.60
$32 – $49	22	13.02
>= $50	22	13.02
*N*	169	

The distribution of consumption reported by participants for each product in the experiment (i.e., outcome) is plotted in S2 Table in S1 Data, showing frequency counts for 0s and different categories of positive units. In this study, study participants on average consumed 3 units per product per choice task, and the average price of products were $7.66.

We estimated own- and cross-price elasticities along with the effects of other attributes (flavor, type, and indoor use) using a zero-inflated negative binomial model, as shown in Table 3. The results show that a 10% increase in beer prices reduced beer consumption by 1% (p < 0.001); a 10% increase in liquor/wine price reduced liquor/wine consumption by 1.1% (p < 0.001); and a 10% increase in cigarette prices reduced cigarette consumption by 1.6% (p < 0.001). While e-cigarette consumption was not significantly impacted by e-cigarette prices, it was significantly impacted by beer and liquor/wine prices. A 10% increase in beer prices raised e-cigarette consumption by 1.25% (p < 0.05) and a 10% increase in liquor/wine prices raised e-cigarette consumption by 0.5%. This finding suggests that e-cigarette demand may increase with increasing prices for alcohol. Other cross-price elasticities were not significant.

**Table 3:** Zero-inflated negative binomial regression: Own- and cross-price elasticities of demand.

*Dependent variable: consumption*	Elasticities (SE)^a^
*Own- and cross-price elasticities*	
*beer consumption in response to:*	
beer prices	-0.103*** (0.021)
liquor/wine price	0.015 (0.016)
e-cigarette price	-0.011 (0.016)
cigarette price	-0.028 (0.024)
*liquor/wine consumption in response to:*	
liquor/wine price	-0.109*** (0.019)
beer price	0.003 (0.023)
e-cigarette price	-0.021 (0.017)
cigarette price	-0.013 (0.026)
*cigarette consumption in response to:*	
cigarette price	-0.164*** (0.026)
e-cigarette price	-0.010 (0.027)
beer price	-0.025 (0.020)
liquor/wine price	-0.013 (0.019)
*e-cigarette consumption in response to:*	
e-cigarette price	-0.035 (0.031)
cigarette price	0.031 (0.031)
beer price	0.125 * (0.055)
liquor/wine price	0.050 * (0.025)
*Tobacco product indoor use*	
no	--
yes	0.154 * (0.063)
*Tobacco product flavors*	
tobacco	--
menthol	-0.071 (0.077)
fruit (e-cigarette only^b^)	-0.207 (0.145)
alcohol (e-cigarette only^b^)	0.046 (0.167)
*Product constants*	
disposable pod e-cigarette	--
rechargeable pod e-cigarette	0.365 * (0.181)
cigarette	2.463*** (0.579)
liquor	2.025*** (0.541)
wine	1.944*** (0.549)
beer	2.338*** (0.534)
*N*	5,131

^a^Elasticities: estimated elasticities and semi-elasticities. SE: standard error. *  *p* <  0.05, ** *p* <  0.01, *** *p* <  0.001. Own- and cross-price elasticities of demand are estimated using Stata command margins, eyex. Semi-elasticities are estimated for other attributes using Stata command margins, eydx. Observations with consumption greater than 50 were dropped, and individual participants without state geo-location, age, family income or employment status information were excluded. We controled for participant age, gender, sexual orientation, race/ethnicity, education attainment, employment status, before-tax family income, participant weekly spending on alcohol and tobacco, and whether the participant used pod e-cigarettes most often. We specified that zero consumption is determined by price, product type, flavor, and whether indoor use is allowed. In addition, we specified a different stepping algorithm to be used in nonconcave regions when maximizing the likelihood function. S4 Table in S1 Data reports the estimated between-subject effects.

^b^Only e-cigarette products have fruit or alcohol flavor options, and cigarette flavors are limited to tobacco or menthol; flavors are not applicable to alcohol products (also see the last column of Table 1).

The results further show that, when both cigarettes and e-cigarettes are allowed to be used indoors, their consumption increased by 15.4% (p < 0.05). In addition, tobacco flavors were not significantly associated with tobacco consumption. Compared to disposable e-cigarettes measured in typical units, participants purchased rechargeables units 36.5% more (p < 0.05) and other product units (cigarettes, beer, liquor/wine) about 2 to 2.5 times more (p < 0.001).

Table 4 shows that if e-cigarettes were allowed to be used indoors at bars and restaurants, e-cigarette purchases increased by 81.2% (*p* <  0.001), compared to if e-cigarette indoor use was not allowed; and when cigarettes were allowed to be used indoor at bars and restaurants, e-cigarette purchases increased by 64.1% (*p* <  0.01). These results suggest that banning e-cigarette and cigarette use indoor will decrease e-cigarette consumption. Whether cigarettes or e-cigarettes are allowed to use indoors did not impact alcohol or cigarette consumption.

**Table 4:** Zero-inflated negative binomial regression: The effects of tobacco indoor use on tobacco and alcohol consumption.

*Dependent variable: consumption*	
	Semi-elasticities (SE)^a^
*Beer consumption in response to:*	
cigarette indoor use allowed	-0.021 (0.075)
e-cigarette indoor use allowed	0.021 (0.075)
*Liquor/wine consumption in response to:*	
cigarette indoor use allowed	0.045 (0.095)
e-cigarette indoor use allowed	-0.116 (0.096)
*Cigarette consumption in response to:*	
e-cigarette indoor use allowed	-0.106 (0.079)
cigarette indoor use allowed	0.054 (0.078)
*E-cigarette consumption in response to:*	
cigarette indoor use allowed	0.641** (0.203)
e-cigarette indoor use allowed	0.812*** (0.189)
*N*	5,131

^a^SE: standard error. ^*^ *p* <  0.05, ^**^ *p* <  0.01, ^***^ *p* <  0.001. Semi-elasticities are estimated using Stata command margins, eydx. Own-price elasticities of demand (not reported in the table) are estimated using Stata command margins, eyex. Observations with consumption greater than 50 are dropped, and individual participants without state geo-location, age, family income or employment status information are excluded. We control for participant age, gender, sexual orientation, race/ethnicity, education attainment, employment status, and before-tax family income, participant weekly spending on alcohol and tobacco, and whether the participant used pod e-cigarettes most often. We specify that zero consumption is determined by price, product type, flavor, and whether indoor use is allowed. In addition, we specify a different stepping algorithm to be used in nonconcave regions when maximizing the likelihood function.

We further tested how the availability of alcohol flavors in e-cigarettes impact the purchases of alcohol and tobacco, as shown in Table 5. Around 15% of study participants reported alcohol-flavored e-cigarettes as the flavor option they used most often, similar to the prevalence of alcohol flavored use among a nationally representative sample of e-cigarette users [35]. The results suggest that the availability of alcohol-flavored e-cigarettes was not significantly associated with alcohol or tobacco purchases.

**Table 5:** Zero-inflated negative binomial regression: The effects of alcohol-flavored e-cigarettes on tobacco and alcohol consumption.

*Dependent variable: consumption*	Semi-elasticities (SE)^a^
*Beer consumption in response to:*	
alcohol flavor available in e-cigarettes	0.103 (0.221)
*Liquor/wine consumption in response to:*	
alcohol flavor available in e-cigarettes	-0.159 (0.533)
*E-cigarette consumption in response to:*	
alcohol flavor available in e-cigarettes	-0.358 (20.315)
*Cigarette consumption in response to:*	
alcohol flavor available in e-cigarettes	-0.086 (0.566)
*N*	5,131

^a^SE: standard error. ^*^ *p* <  0.05, ^**^ *p* <  0.01, ^***^ *p* <  0.001. Semi-elasticities are estimated using Stata command margins, eydx. Own-price elasticities of demand (not reported in the table) are estimated using Stata command margins, eyex. Observations with consumption greater than 50 are dropped, and individual participants without state geo-location, age, family income or employment status information are excluded. We control for participant age, gender, sexual orientation, race/ethnicity, education attainment, employment status, before-tax family income, participant weekly spending on alcohol and tobacco, and whether the participant used pod e-cigarettes most often. We further specify that zero consumption is determined by price, product type, flavor, and whether indoor use is allowed. In addition, we specify a different stepping algorithm to be used in nonconcave regions when maximizing the likelihood function.

In addition to attributes that were manipulated in the experiment, the regressions also controlled for between-subject differences, such as weekly spending and current use status. As shown in S4 Table in S1 Data, weekly spending on alcohol or tobacco increased the purchases of all products (*p* <  0.001), controlling for participant demographics. Relative to cisgender male participants, cisgender female participants reported buying more (*p* <  0.001), controlling for other demographic characteristics; and non-Hispanic Black or African American participants (*p* <  0.001) were buying fewer units relative to non-Hispanic White participants. Participants with higher educational attainment were generally buying fewer units compared with those who completed 12th grade or less with no high school diploma, though not always statistically significant. Compared to participants who reported currently working, those who were looking for work or unemployed (*p* <  0.05), and who were disabled permanently or temporarily (*p* <  0.05) were buying fewer units, controlling for family income level and other socio-demographics as shown in S4 Table in S1 Data. Individual participants with family income between $25,000 - $49,999 (*p* <  0.001) and those with family income of $50,000 and above (*p* <  0.01) were buying fewer units relative to participants with less than $25,000 family income, controlling for other demographic characteristics. Sensitivity analyses (S5 Table in S1 Data) generally demonstrated that higher tax burdens are associated with lower consumption, with 75% tax burden having the greatest impact on reducing consumption.

## Discussion

Smoking and excessive alcohol consumption are known risks for cancer, and alcohol and tobacco are consumed concurrently to a high degree [64,65]. Therefore, regulating nicotine/tobacco products may have spillover effects on alcohol drinking and vice versa. There is a great opportunity to optimize the public health benefits of tobacco and alcohol regulatory policies through coordinated actions that take into account cross-product policy impacts (i.e., how the regulation of one product impacts the consumption of another) [66].

Although several studies have estimated cross-price elasticities between cigarettes and e-cigarettes [46–50,67–69], and between cigarettes and beer [70,71], there has been limited experimental evidence to study multiple tobacco and alcohol products systematically [67]. This study is the first to our knowledge to study cross-product policy impacts among cigarettes, e-cigarettes, liquor/wine, and beer, including pricing (taxation), place regulation (indoor use laws), and flavor restrictions. The evidence from this study can help policymakers by considering multiple products simultaneously and taking into account of cross-product policy impacts beyond cigarette-e-cigarette price elasticities or cigarette–beer price elasticities that have been studied previously [4,14].

Consistent with existing literature and the law of demand, we found that increasing prices through raising tax burdens significantly reduce the consumption of cigarettes, beer, and liquor/wine. Specifically, we found price elasticities for cigarette, beer, and liquor/wine consumption to be -0.2, -0,1, -0.1, respectively. The cigarette price elasticity estimate is within the range of elasticity estimates (-0.2 to -0.6) reported for the US by the International Agency for Research on Cancer (IARC) review, while alcohol price elasticity estimates [72] are smaller than the existing systematic review estimates, which show the aggregated elasticity to be -0.17 for beer and -0.3 for wine/liquor [73,74]. It is possible that cigarette and alcohol users who recently initiate e-cigarettes are less price responsive to alcohol prices than the rest of the population. In addition, VCEs mostly rely on within-subject price variation to estimate price elasticities [51], which may explain why we found lower elasticities compared to prior research, which uses observational data and mostly between-subject price variation to estimate price elasticities. Moreover, in a VCE, participants respond to hypothetical questions, whereas observational studies use revealed preference data [75]; however, stated preferences derived from choice experiments can adequately predict behavioral responses in real-world settings [76].

Prior research using retail sales and survey data found that e-cigarette sales and use prevalence were responsive to price changes [46–49,69,77–83]. In our study, we found negative but non-significant impact of e-cigarette prices on consumption. Sensitivity analyses further showed that while a 75% tax burden significantly decreases e-cigarette consumption, a 25% tax burden was positively associated with consumption and a 50% tax burden was not significantly impacting consumption. Unlike previous studies, we examined a unique sample of participants who co-use cigarettes and alcohol and recently started using e-cigarettes. As they were new e-cigarette users, they may not have been as sensitive to e-cigarette prices as established users would, especially when tax burdens and price levels are not as high as a 75% tax burden. In addition, they may try e-cigarettes to complement their use of cigarettes or to help them with quitting or reduce harms of smoking, which explains why their responsiveness to e-cigarette prices are not linear and may prefer slightly higher prices with a 25% tax burden for quality. Importantly, e-cigarettes is a very heterogenous group of products and in some cases, increased price on e-liquid, can be compensated for to some degree by adjusting the e-cigarettes, and e-cigarette devices have varying nicotine yield depending on device power, nicotine strength and form in e-liquids, user puffing behaviors, and so on [84,85].

Our results further suggest that e-cigarettes are economic substitutes to beer and liquor/wine for adult smokers who use multiple substances. A 10% increase in beer or alcohol prices will increase e-cigarette consumption among this group by 1.25% and 0.5%, respectively. This evidence provides support to increase taxes on alcoholic beverages, which has been declining since 1970 due to infrequent tax increases and inflation erosion [86]. Moreover, given that e-cigarettes are considered a less harmful alternative to cigarettes, and if smokers initiate or re-initiate e-cigarette use and eventually completely switch to e-cigarettes (and quit smoking), that may be beneficial to them from a harm reduction perspective [87]. Therefore, increasing alcohol prices have the potential to lead to harm reduced tobacco use among this group.

One concern with taxing e-cigarettes is that it may increase cigarette smoking [46,47,49], leading to unintended consequences. Our study shows that at least among adults using all three substances (cigarettes, e-cigarettes and alcohol), e-cigarette taxes did not increase smoking or alcohol drinking among this group (the cross-price elasticities of how e-cigarette taxes impact cigarette and alcohol consumption were non-significant). Therefore, increasing both alcohol and e-cigarette taxes simultaneously may have the desired policy effect of encouraging adult smokers who use multiple substances to consume more e-cigarettes while reducing alcohol use.

In addition to pricing, whether nicotine/tobacco products are banned or allowed for indoor use is another important policy area. Since alcohol-serving facilities such as bars and restaurants are one of the primary targeted locations for smoke- or vape- free air regulations, it is important to know how these tobacco policies may impact alcohol use. Previous research has shown that smoking bans in bars and restaurants can significantly decrease the drinking intensity among smokers [88]. In this study, we found that for adults who use multiple substances, their purchases of alcoholic beverages did not respond to whether cigarettes or e-cigarettes are allowed/banned indoors. It is possible that smoke-free indoor places may have become a norm as a consequence of the increasing implementation of comprehensive smoke-free air laws. Therefore, the impact of such laws on alcohol consumption may have become non-significant. In addition, as the participants are established cigarette and alcohol co-users, they may not respond to tobacco indoor policies by adjusting alcohol use.

Our findings further suggest that if e-cigarettes or cigarettes were allowed to be used indoors, the purchases of e-cigarettes would increase regardless. It is not surprising that when e-cigarettes are allowed to be used indoors, their consumption would increase. However, as a potential substitute for cigarettes, e-cigarette consumption increases even when cigarettes are allowed to be used indoors. This could be again explained by sample characteristics, especially participants’ recent initiation of e-cigarette use. They may use e-cigarettes for their less perceived harm for bystanders and themselves or are in the trajectories of consuming more e-cigarettes, therefore not considering whether they can be substitutes for cigarettes indoors or not.

Tobacco and alcohol use also intersects when tobacco products are characterized as alcohol flavored. Although the 2009 Family Smoking Prevention and Control Act (FSPTCA) has banned flavored cigarettes except for those with mint-, menthol, or tobacco flavors, alcohol flavors are still available in nicotine or tobacco products other than cigarettes. There is strong evidence that links flavors with youth initiation of nicotine or tobacco products [89,90]. However, attractive flavors may also play a role in motivating adult smokers to try less harmful products such as e-cigarettes [87]. Therefore, how flavors in tobacco or nicotine tobacco products should be regulated has stirred a heated debate in recent years.

Nonetheless, the US Food and Drug Administration (FDA) and state and local governments have increasingly restricted flavors in nicotine or tobacco products, including certain e-cigarette models (e.g., open system e-cigarettes with flavors other than menthol/tobacco/mint). Moreover, the FDA has requested premarket tobacco product applications (PMTA) for e-cigarette products on the market after February 2007 and used flavors as a criterion to disapprove nearly 1 million e-cigarette flavored products [91]. As of July 18, 2024, the FDA had authorized 34 e-cigarette products and devices, including 4 non-tobacco flavored e-cigarette products [92,93], yet many non-tobacco flavored e-cigarettes without authorizations remained available on the market [94].

In this study, we explicitly examined whether the availability of alcohol flavors in e-cigarette products (policy scenarios where alcohol-flavored e-cigarettes are legally sold in the market and available for purchases) impacted alcohol or cigarette use among adults who use multiple substances. The results suggest that alcohol flavors in e-cigarettes did not have a significant impact on alcohol purchase or tobacco purchases. This is likely because while alcohol flavors were common in e-cigarettes, the most appealing or prevalent flavors fact fruit or sweet flavors [36]. Prior studies that examine how flavors in cigars and cigarillos impact product preference also suggest that fruit flavors are the most preferred, and there was no significant difference in the preference for wine flavor over tobacco or menthol flavors [32]. Therefore, for adults who use multiple substances (e-cigarettes, cigarettes, and alcohol), it is unlikely that prohibiting alcohol flavors in e-cigarettes will lead to any significant behavioral changes.

We also found that participants with higher family income were buying fewer units in this experiment. Prior research showed that people with higher socioeconomic status (SES) tend to consume more or equal units of alcoholic beverages relative to those with lower SES [95], while higher SES is associated with less tobacco consumption [96,97]. It is important to note the uniqueness of our sample, and that adult smokers who reported current alcohol use and were at the stage of recent e-cigarette (re-)initiation may have different preferences for tobacco and alcohol compared to other populations (e.g., exclusive smokers who never used cigarettes), and thus cautions should be taken before generalizing the study results.

This study has several limitations. First, stated preferences are imperfect indicators of individual persons’ real-world choices, though existing evidence demonstrates the correspondence between stated preference data and people’s actual behaviors; moreover, stated preferences are particularly useful for evaluating hypothetical policies [62,75,98]. Second, we recruited a convenience sample which might not be representative of US adult smokers who recently (re)initiated e-cigarette use and also consumed alcohol in the past 30 days. For instance, cisgender white women appear to be over-represented in the study sample. However, the sample of adult smokers who use both e-cigarettes and alcohol is difficult to recruit, and few studies have examined concurrent users of cigarettes and e-cigarettes near the point of e-cigarette initiation. Furthermore, our study presents crucial evidence about this population as the sample was recruited nationally. Third, the study data was collected during early to middle stage of the COVID-19 pandemic, which might have altered alcohol and tobacco preferences of individual persons.

## Conclusions

Based on VCEs, findings from our study suggest that for adult smokers who also drink alcohol and use e-cigarettes, increasing prices on these products will not lead to unintended consequences. Moreover, increasing alcohol prices may incentivize smokers to purchase more e-cigarettes, which has the potential to motivate complete transitions from cigarette smoking to e-cigarette use among this group. If tobacco use is allowed indoors, this group of adult substance users might purchase more e-cigarettes. The availability of alcohol flavor in e-cigarettes did not have any impacts on adult smokers who also drink and use e-cigarettes. Policymakers may consider this evidence when designing policy regulations over multiple substances.

## Supporting information

S1 Data S1 File. Instructions to participants about VCEs. S2 Table. Distribution of consumption units reported by study participants for each product. Observations with over 50 consumption units are removed from the analysis. S3 Table. Summary statistics of participant demographics. S4 Table. Zero-inflated negative binomial regressions: Own- and cross-price elasticities of demand, and the effects of tobacco control policies on alcohol consumption (between-subject effects). S5 Table. Zero-inflated Poisson regression: Sensitivity analysis estimating changes in consumption in response to different levels of tax burdens.
(DOCX)
