# Correction: The impacts of product characteristics and regulatory environment on smokers’ preferences for tobacco and alcohol: Evidence from a volumetric choice experiment

**DOI:** 10.1371/journal.pone.0339046

**Published:** 2025-12-15

**Authors:** Shaoying Ma, Ce Shang, Vuong V. Do, Jidong Huang, Terry F. Pechacek, Scott R Weaver

In the Methods subsection of the Abstract, there is an error in the paragraph. The correct paragraph is: A total of 182 US adult smokers who were using e-cigarettes and consuming alcohol participated in online volumetric choice experiments and reported on the quantity they would purchase among cigarettes, closed-system e-cigarettes, beer, and one other alcohol product (wine/liquor) under varying policy scenarios.

In the Analysis subsection of the Materials and methods, there is an error in the third sentence of the sixth paragraph. The correct sentence is: We controlled for the following demographic variables in all specifications: age, gender, sexual orientation, race/ethnicity, education, employment status, and total family income (in 2019 before taxes). Participants with missing age (n = 1), before-tax family income in 2019 (n = 2), employment status (n = 1), or state of residence (n = 2) were removed from the analysis. In addition, 17 observations with extreme values of consumption (greater than 50 units) and 7 participants with extreme or missing consumption responses in all choice questions were also excluded. There were 5,131 observations from 169 participants remaining in the analytic sample. Choice experiments provide repeated measures from each participant, and thus our VCE study is designed to detect small effect sizes.

S1 Data is incorrect. It can be viewed below.

## Supporting information

S1 Data (DOCX)
