# Supplementary material for: The impacts of product characteristics and regulatory environment on smokers’ preferences for tobacco and alcohol: Evidence from a volumetric choice experiment
Source: PLoS One. 2025 Mar 12;20(3):e0320023. doi: 10.1371/journal.pone.0320023 (PMC11902124; doi:10.1371/journal.pone.0320023)
Supplement: S1 Data — S1 File. Instructions to participants about VCEs. S2 Table. Distribution of consumption units reported by study participants for each product. Observations with over 50 consumption units are removed from the analysis. S3 Table. Summary statistics of participant demographics. S4 Table. Zero-inflated negative binomial regressions: Own- and cross-price elasticities of demand, and the effects of tobacco control policies on alcohol consumption (between-subject effects). S5 Table. Zero-inflated Poisson regression: Sensitivity analysis estimating changes in consumption in response to different levels of tax burdens. (DOCX) [file pone.0320023.s001.docx]

**S1. Instructions to participants about VCEs**

Next, we will show you 8 different store shelf displays for tobacco and alcohol products. You have seen similar questions in the last survey. Some details are different here. So, please read carefully.

We are interested in your purchase and consumption of tobacco and alcohol. For alcohol, this includes purchases that you would make both in stores and at bars or restaurants. There are no right or wrong answers. The only thing that matters is that you try your best to tell us what you would actually do if the products below were available the next time you shop for tobacco or alcohol.

For each product, we will provide more information about:

- Type of tobacco or alcohol product
- Price
- Tobacco flavor:
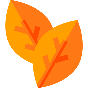
, menthol
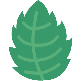
, fruit
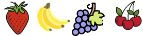
, or alcohol
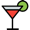

- Whether the tobacco product can be used in restaurants and bars

You will be asked to evaluate the products offered in each shelf display and tell us how many of each you would be likely to buy the next time you shop for tobacco or alcohol. All you need to do is enter a number for each product to tell us how many you want to buy and use in a TYPICAL WEEK, **if these were your only options**.

If you buy tobacco or alcohol in larger sizes (carton of cigarettes or bottle of wine), please do your best to convert to the number of individual units that you would **buy and consume** in a typical week. A typical bottle of wine equals 5 glasses, and a 750 ml bottle of liquor equals about 17 1.5 oz. shots.

If you would buy none of a product, please enter zero. If you would buy none of any product in an entire store shelf, you may enter zero for all products OR check “I would not buy ANY of these products”.

**Assume** that you would use the products in a TYPICAL WEEK (you cannot save, share, sell, or stockpile for a later date). Your typical weekly budget for buying tobacco and alcohol products is shown. You do not have to stick to this budget—but consider it as you make your choices.

**S2. Distribution of consumption units reported by study participants for each product** (observations with over 50 consumption units are removed from the analysis)

| **S3. Summary Statistics of Participant Demographics** | | |
| --- | --- | --- |
|  | Mean | SD |
| *Participant age* | 38 | 10.20 |
|  | Number of participants | Percent |
| *Gender* |  |  |
| Cisgender male | 57 | 33.73 |
| Cisgender female | 108 | 63.91 |
| Transgender, non-binary, and other gender minorities | 4 | 2.37 |
| *Sexual orientation* |  |  |
| Gay | 5 | 2.96 |
| Lesbian | 7 | 4.14 |
| Straight | 132 | 78.11 |
| Bisexual | 20 | 11.83 |
| Other sexual minoritized groups | 5 | 2.96 |
| *Race/ethnicity* |  |  |
| American Indian or Alaska Native | 3 | 1.78 |
| Non-Hispanic Black or African American | 22 | 13.02 |
| Asian | 1 | 0.59 |
| Native Hawaiian or Pacific Islander | 2 | 1.18 |
| Non-Hispanic White | 117 | 69.23 |
| Non-Hispanic other race | 1 | 0.59 |
| Non-Hispanic, multi-race | 6 | 3.55 |
| Hispanic | 17 | 10.06 |
| *Education attainment* |  |  |
| 12th grade or less, no diploma | 5 | 2.96 |
| High school graduate | 32 | 18.93 |
| GED or equivalent | 16 | 9.47 |
| Some college, no degree | 63 | 37.28 |
| Associate degree | 21 | 12.43 |
| Bachelor's degree (e.g., BA, AB, BS, BBA) | 27 | 15.98 |
| Master's degree (e.g., MA, MS, MEng, MEd, MBA) | 4 | 2.37 |
| Professional school degree (e.g., MD, DDS, DVM, JD) | 1 | 0.59 |
| *Employment status* |  |  |
| Working now | 84 | 49.70 |
| Only temporarily laid off, sick leave, or maternity leave | 5 | 2.96 |
| Looking for work, unemployed | 32 | 18.93 |
| Retired | 4 | 2.37 |
| Disabled permanently or temporarily | 17 | 10.06 |
| Keeping house | 18 | 10.65 |
| Student | 4 | 2.37 |
| Other | 5 | 2.96 |
| *Family income* |  |  |
| Less than $25,000 | 60 | 35.50 |
| $25,000 - $49,999 | 51 | 30.18 |
| $50,000 and above | 58 | 34.32 |
| *N* | 169 |  |

SD: standard deviation.

**S4. Zero-inflated negative binomial regressions: own- and cross-price elasticities of demand, and the effects of tobacco control policies on alcohol consumption (between-subject effects)**

| *Dependent variable: consumption* | Coef. (SE) |
| --- | --- |
| age | -0.003 (0.002) |
| gender |  |
| cisgender male | -- |
| cisgender female | 0.179^***^ (0.045) |
| transgender, non-binary, and other gender minorities | -0.551^**^ (0.184) |
| sexual orientation |  |
| straight | -- |
| gay | 0.408^**^ (0.125) |
| lesbian | 0.153 (0.104) |
| bisexual | -0.014 (0.062) |
| other sexual minorities | -0.039 (0.122) |
| race/ethnicity |  |
| Non-Hispanic White | -- |
| Non-Hispanic Black or African American | -0.438^***^ (0.058) |
| American Indian or Alaska Native | -0.385^**^ (0.121) |
| Asian | -0.674^*^ (0.269) |
| Native Hawaiian or Pacific Islander | -0.243 (0.165) |
| Non-Hispanic other race | 0.766^**^ (0.282) |
| Non-Hispanic, multi-race | 0.156 (0.100) |
| Hispanic | 0.002 (0.066) |
| education attainment |  |
| 12th grade or less, no diploma | -- |
| high school graduate | -0.298^*^ (0.130) |
| GED or equivalent | 0.055 (0.134) |
| some college, no degree | -0.356^**^ (0.124) |
| associate degree | -0.380^**^ (0.133) |
| bachelor's degree (e.g., BA, AB, BS, BBA) | -0.164 (0.131) |
| master's degree (e.g., MA, MS, MEng, MEd, MBA) | -0.859^***^ (0.165) |
| professional school degree (e.g., MD, DDS, DVM, JD) | -0.157 (0.255) |
| employment status |  |
| working now | -- |
| only temporarily laid off, sick leave, or maternity leave | 0.184 (0.105) |
| looking for work, unemployed | -0.163^**^ (0.056) |
| retired | -0.220 (0.137) |
| disabled permanently or temporarily | -0.154^*^ (0.067) |
| keeping house | 0.093 (0.061) |
| student | 0.070 (0.134) |
| other | -0.173 (0.114) |
| family income |  |
| less than $25,000 | -- |
| $25,000 - $49,999 | -0.191^***^ (0.050) |
| $50,000 and above | -0.158^**^ (0.050) |
| weekly spending on alcohol | 0.015^***^ (0.001) |
| weekly spending on tobacco | 0.002^***^ (0.000) |
| participant used pod e-cigarettes most often | -0.019 (0.039) |

The above are estimated coefficients of variables as regressors of positive consumption. Estimated within-subject effects (attributes) are reported in Table 3. ^*^ *p* < 0.05, ^**^ *p* < 0.01, ^***^ *p* < 0.001. SE: standard error.

**S5. Zero-inflated Poisson regression: Sensitivity analysis estimating changes in consumption in response to different levels of tax burdens.**

| *Dependent variable: consumption* | Semi-elasticities (SE)^a^ |
| --- | --- |
| *Own elasticities* |  |
| *beer consumption in response to:* |  |
| 0% tax burden | -- |
| 25% tax burden | -0.116 (0.065) |
| 50% tax burden | -0.293^***^ (0.064) |
| 75% tax burden | -0.869^***^ (0.150) |
| *liquor/wine consumption in response to:* |  |
| 0% tax burden | -- |
| 25% tax burden | -0.102 (0.075) |
| 50% tax burden | -0.390^***^ (0.073) |
| 75% tax burden | -0.779^***^ (0.096) |
| *cigarette consumption in response to:* |  |
| 25% tax burden | -- |
| 50% tax burden | -0.107 (0.067) |
| 75% tax burden | -0.625^***^ (0.072) |
| *e-cigarette consumption in response to:* |  |
| 0% tax burden | -- |
| 25% tax burden | 0.231^**^ (0.086) |
| 50% tax burden | -0.046 (0.146) |
| 75% tax burden | -0.497^***^ (0.099) |
| *Tobacco product indoor use* |  |
| no | -- |
| yes | 0.193^***^ (0.044) |
| *Tobacco product flavors* |  |
| tobacco | -- |
| menthol | -0.063 (0.049) |
| fruit (e-cigarette only^b^) | 0.151 (0.081) |
| alcohol (e-cigarette only^b^) | -0.074 (0.106) |
| *Product constants* | |
| disposable pod e-cigarette | -- |
| rechargeable pod e-cigarette | 0.189^**^ (0.067) |
| cigarette | 1.141^***^ (0.097) |
| liquor | 1.094^***^ (0.107) |
| wine | 0.998^***^ (0.108) |
| beer | 1.334^***^ (0.101) |
| *N* | 5,131 |

^a^SE: standard error. ^*^ *p* < 0.05, ^**^ *p* < 0.01, ^***^ *p* < 0.001. Semi-elasticities are estimated using Stata command margins, eydx. Observations with consumption greater than 50 are dropped, and individual participants without state geo-location, age, family income or employment status information are excluded. We control for participant age, gender, sexual orientation, race/ethnicity, education attainment, employment status, before-tax family income, participant weekly spending on alcohol and tobacco, and whether the participant used pod e-cigarettes most often. We specify that zero consumption is determined by tax burdens, product type, flavor, and whether indoor use is allowed. In addition, we specify a different stepping algorithm to be used in nonconcave regions when maximizing the likelihood function.

^b^Only e-cigarette products have fruit or alcohol flavor options, and cigarette flavors are limited to tobacco or menthol; flavors are not applicable to alcohol products (also see the last column of Table 1).
